# Thermal Transport of Graphene Sheets with Fractal Defects

**DOI:** 10.3390/molecules23123294

**Published:** 2018-12-12

**Authors:** Yang Kang, Fuyan Duan, Shaoxin Shangguan, Yixin Zhang, Tianpei Zhou, Bingcheng Si

**Affiliations:** College of Water Resources and Architectural Engineering, Northwest A&F University, Yangling 712100, China; duanfuyan@nwafu.edu.cn (F.D.); shaoxinshangguan@nwafu.edu.cn (S.S.); zhangyixin@nwafu.edu.cn (Y.Z.); zhoutp@nwafu.edu.cn (T.Z.)

**Keywords:** molecular dynamics, graphene, fractal defects, thermal conductivity

## Abstract

Graphene combined with fractal structures would probably be a promising candidate design of an antenna for a wireless communication system. However, the thermal transport properties of fractal graphene, which would influence the properties of wireless communication systems, are unclear. In this paper, the thermal transport properties of graphene with a Sierpinski fractal structure were investigated via the reverse non-equilibrium molecular dynamics simulation method. Simulation results indicated that the thermal conductivity of graphene with fractal defects decreased from 157.62 to 19.60 (W m^−1^ K^−1^) as the fractal level increased. Furthermore, visual display and statistical results of fractal graphene atomic heat flux revealed that with fractal levels increasing, the real heat flux paths twisted, and the angle distributions of atomic heat flux vectors enlarged from about (−30°, 30°) to about (−45°, 45°). In fact, the fractal structures decreased the real heat flow areas and extended the real heat flux paths, and enhanced the phonon scattering in the defect edges of the fractal graphene. Analyses of fractal graphene thermal transport characters in our work indicated that the heat transfer properties of fractal graphene dropped greatly as fractal levels increased, which would provide effective guidance to the design of antennae based on fractal graphene.

## 1. Introduction

The antenna is the fundamental component of transmitting and receiving radio waves in wireless communication systems, which restricts the performance and size of the wireless communication system. In order to minimize the size of the antenna and make effective use of valuable spectrum resources, it is appropriate to find better antenna materials and antenna structures in comparison to traditional microstrip antennae based on Euclidean geometry. Graphene has a unique two-dimensional planar structure, i.e., tunable thermal and electric conductivity [1], which provides many potential engineering applications in the realm of wireless communication systems, including high-performance antennae [2,3,4,5,6]. Additionally, the surface impedance of graphene can be adjusted macroscopically by applying bias voltage [3,4,5]. Moreover, the high electrical resistance of graphene in terahertz band can inhibit the radiation side lobe of the antenna, which enables the graphene antenna to achieve directional radiation and improve the gain [3]. 

Fractal geometry [7], whose perimeter becomes larger and larger, while the area becomes smaller and smaller with fractal level increasing, can be applicable to the design of an antenna structure [8]. Compared to traditional microstrip antennae, because of its unique self-similarity geometry, the fractal antenna has two obvious advantages. Firstly, the multifrequency performance of the electrical parameters can be obtained. Secondly, the space filling of the fractal structure makes it possible to reduce the size of some antennae [9]. Therefore, fractal graphene may be an effective technical approach for high-performance multiband antennae with a small size. Notably, the excessive power and overheating of an electronic device will affect its service performance and lifetime. When the heat inside a high-power device cannot be released in time, the heat inside the device will cause heat accumulation and deteriorate the reliability of the device [10]. Therefore, an antenna should take thermal transport into account to increase the reliability of the communication system. That is, it is necessary to study the thermal transportation properties of fractal graphene. Many advances in the fields of nanoelectronics and thermal management [11,12] have been achieved ever since the pioneering work of Novoselov et al. [13]. The thermal conductivity of the graphene monolayer can be up to 5300 *W*·*m*^−1^·*K*^−1^ [3,11,14,15] at room temperature. Because of the strong covalent bond between carbon and carbon, graphene conducts heat through lattice vibration, i.e., phonon. Molecular dynamics (MD) is an appropriate method to study phonon vibration, which dominates the thermal transport of graphene. The thermal features of graphene sheets have been regulated through chemical doping [16] and physical tailoring [17]. However, graphene sheets with fractal defects have seldom been studied [18,19,20]. 

In this work, graphene with Sierpinski carpet fractal models with different fractal levels are established, and their thermal conductivity and interfacial thermal resistances are calculated using the reverse nonequilibrium molecular dynamics method (RNEMD) method [21]. Furthermore, the microheat flux on each atom of these fractal graphene sheets is calculated and compared with perfect graphene to verify the microscopic thermal transportation mechanism. 

## 2. Model Structure

**Figure 1:**
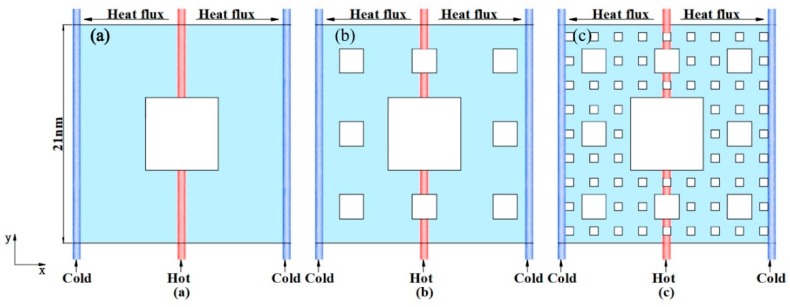
Structure schematics of graphene with different fractal levels (**a**) *n* = 1 (gra1); (**b**) *n* = 2 (gra2); (**c**) *n* = 3 (gra3).

The Sierpinski fractal structure is one of self-similar sets. Generally, a self-similar set is fabricated by the technique of subdividing a shape into smaller copies of itself, removing one or more copies, and continuing recursively, which can be extended to other shapes. Additionally, the construction of the Sierpinski carpet begins with a square. The generation process of the Sierpinski fractal structure is as follows: Firstly, a square is cut into 9 congruent subsquares in a 3-by-3 grid, and the central subsquare is removed. The fractal level 1 pattern is shown in Figure 1a. The same procedure is then applied continuously twice to the remaining 8 subsquares. The self-similar fractal dimension D of the fractal structure is calculated by:

(1) $$D=\frac{\mathrm{lg}b}{\mathrm{lg}a}$$

where *a* is the number of side lengths divided equally and *b* is the ratio of the remaining area of removing the central square to the area of the square after the n*^th^* order. The self-similar fractal dimension of the fractal structure constructed in this paper is of 1.89, according to Equation (1).

## 3. Molecular Dynamics Simulation

In this paper, graphene models with different fractal levels of 1, 2, and 3, and perfect graphene (gra0) have been established and studied. The chirality and size of each model are exactly the same. The chirality is Ziazag, and the side length is 21 nm. The thermal properties of graphene with different fractal levels were simulated using the RNEMD method [21]. As shown in Figure 1, in this work, the heat flux was imposed along the x direction to generate a temperature gradient through the Nosé–Hoover thermal bath [22,23]. The interaction between two carbon atoms was described by the adaptive intermolecular reactive empirical bond-order (AIREBO) potential [24], which has been widely used to study the thermal and mechanical properties of graphene nanomaterials [18,25]. The simulating time step was set to be of 0.5 fs. Both the in-plane and out-plane directions were used for the periodic boundary condition. The dimension of the out-plane simulation box was set to be from –1 nm to 1 nm. Additionally, the monolayer graphene was placed in the origin position of the out-of-plane direction to avoid interaction between replicas of the monolayer graphene. Molecular dynamics were conducted using the large-scale atomic/molecular massively parallel simulator (LAMMPS) [26]. Firstly, the energy of the system was minimized using the conjugate gradient method [27]. Then, simulations were carried out in a NVT (constant atom number, volume, and temperature) ensemble at 300 K after 200 ps, which was long enough to reach an initial equilibrium for the system. The system was later switched to a NVE (constant atom number, volume, and energy) ensemble for 4 ns, of which 1 ns was to obtain a nonequilibrium steady state and then, 3 ns was used to produce an average temperature profile. The thermal conductivity of the graphene with fractal defects was calculated using RNEMD. The entire structural models were divided into 50 slabs along the x direction, where the 1st slab and the 50th slab were cold regions, and the 26th was a hot region. The hottest atom in the cold region periodically swaps its energy with the coldest atom in the hot region in order to achieve a temperature gradient. Here, the heat flux *J_x_* can be calculated by:

(2) $$J_{x}=\frac{\sum_{N_{swap}}\frac{1}{2}(mv_{s}^{2}-mv_{h}^{2})}{t_{swap}}$$

*J_x_* is heat flux; *N_swap_* is the pair number of the atoms involved in exchange of kinetic energy in the simulation process; *t_swap_* means the total time of exchanging atomic energy in the whole simulation process; and *v_s_* and *v_h_* are the velocity of the hottest atoms in the heat sink region and the coldest atoms in the heat source region, respectively. The energy was exchanged every 100 steps and the heat flow from the heat source to the heat sink reached a steady state after 1.0 ns. Then, a stable temperature gradient appeared in the model. The temperature for each slab was computed from:

(3) $$T_{i}(slab)=\frac{2}{3Nk_{B}}\sum_{j}\frac{p_{j}^{2}}{2m}$$

*T*_i_ is the temperature of the i*^th^* slab, N is the number of atoms contained in the i*^th^* slab, *k_B_* is the Boltzmann constant, and $p_{j}$ is the momentum of the *j* atom. 

**Figure 2:**
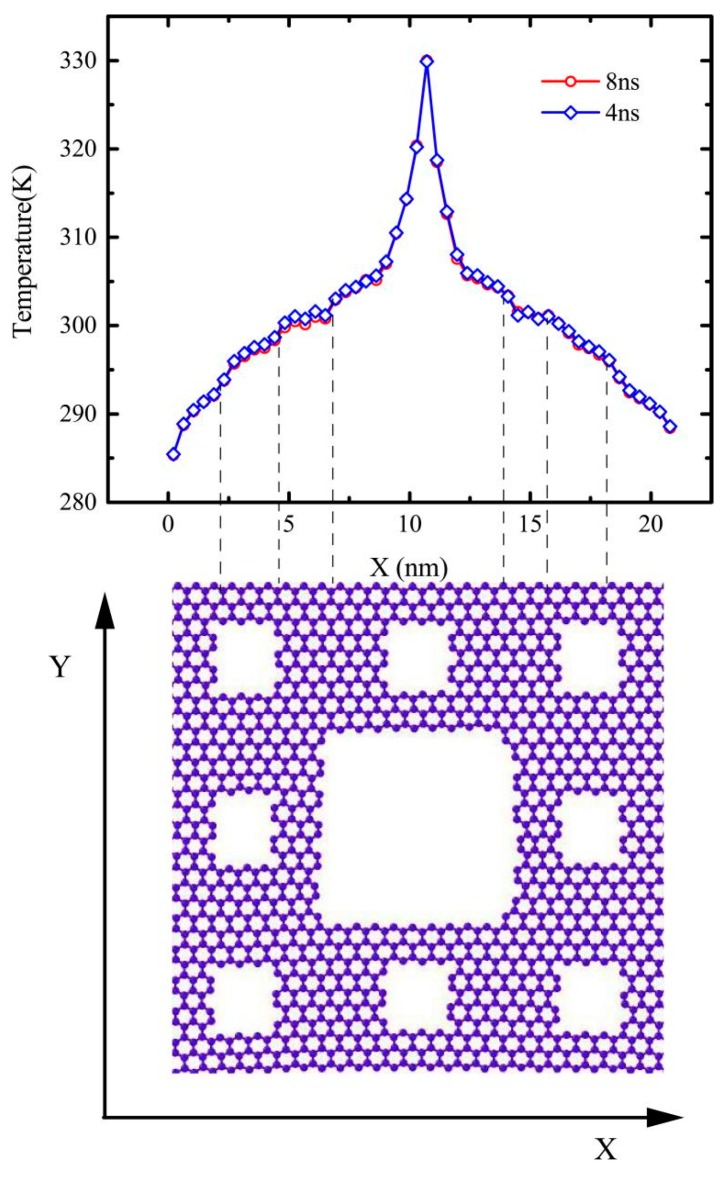
The temperature profile of the gra2 running 8 ns and 4 ns in the NVE ensemble.

**Figure 3:**
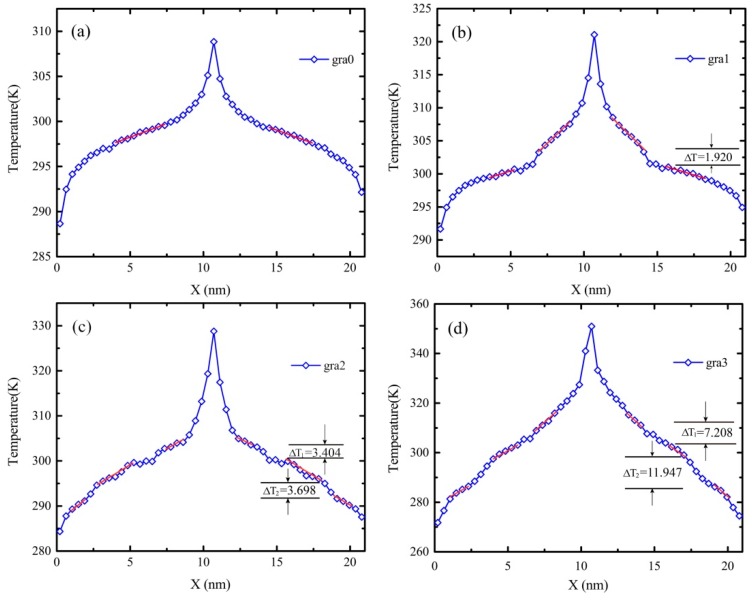
The schematic of plot statistical means temperature distribution of graphene along a zigzag direction. (**a**–**d**) represent the gra0, gra1, gra2, and gra3, respectively.

During energy exchange, the linear response of the temperature variation happens beyond the defective area, where it means interfacial thermal resistance appears. The temperature differences at jumping points, ∆T, as shown in Figure 2 and Figure 3, can be obtained by linearly fitting the temperature variation in each slab. In addition, the temperature distribution is linear in the central region between the heat source and heat sink, which is consistent with the temperature distribution obtained by Fourier’s heat conduction law. The thermal conductivity *κ* can be calculated by the following formula:

(4) $$\kappa =\frac{J_{x}}{2A\partial T/\partial x}$$

where *A* represents the cross-sectional area through the heat flux, which can be described by the width of the model to multiply the thickness of the graphene sheet. We took the thickness of the graphene sheet as 0.142 nm, the carbon–carbon bond length, which was widely adopted to calculate the thermal conductivity of graphene sheet and nanotube [18].

The interfacial thermal resistance, also known as the Kapitza resistance, has been defined as the ratio of the temperature difference and the heat flux across the interface:

(5) $$R=\frac{\Delta T}{J}$$

where ∆*T* represents the temperature difference and *J* is constant heat flux. 

## 4. Results and Discussions

The temperature profile of gra3 seems smoother than that of the gra2, as shown in Figure 3c,d. The reason for the roughness of the temperature profile of the gra2 may be fractal defects, which inherently affect the heat flux transfer or the insufficient time to average temperature. Therefore, we supplemented a simulation about the gra2 in same condition except that the system is running for 8 ns in a NVE ensemble, of which the 1st 4 ns that were enough to reach a nonequilibrium steady state and the 2nd 4 ns was used to average the temperature profile. As shown in Figure 2, there is almost no difference between the temperature profiles with 8 ns and 4 ns. Therefore, the unsmoothness of temperature profiles was attributed to the fractal defects with different levels. Then, we continued this work in terms of the 4ns’ simulation time, which is sufficient to obtain accurate results.

As shown in Figure 3, in agreement with the previous study [28], drops were observed in the temperature profile due to the space vacancy, and they indicated the presence of interfacial thermal resistance. The jump in the temperature profile is associated with the reduction of the heat flux. Moreover, the temperature jumps near the low temperature side are larger than those near the high temperature side, which is consistent with the conclusion of a rectangular defect graphene structure [20]. In order to decrease the error, the temperature jump values were the differences of the linear fitting data at the jump points. As shown in Figure 3, the temperature profile of gra3 is smoother than the result of gra2. Unexpectedly, the temperature jumps of gra3 varied greatly at the same position as gra2 and the corresponding interfacial thermal resistance effect of gra3 is more obvious than those of gra2, as shown in the Table 1. Generally, temperature jumps of a greater fractal level are bigger than those of lower fractal levels, as shown in Figure 3 and Table 1. That is, the biggest temperature drop occurs in the boundary or edge near defects of gra3. It seems that thermal carriers are trapped and energy is accumulated in these edge regions, but the exact physical images of heat transport at the atomic level are not clear.

In addition, the original temperatures of the systems are set to be 300 K and the energy of all simulation systems is identical. When the system is in the steady state, the stable temperature difference between heat resource and heat sink increases as the fractal level raises, which is shown in Figure 3. The temperature difference of gra0 is 20 K and that of gra3 is 82 K. According to Equation (3), this phenomenon can be attributed to the decrease of the atom numbers of fractal graphene as the fractal level increases, while the momentum of all models is congruent.

**Figure 4:**
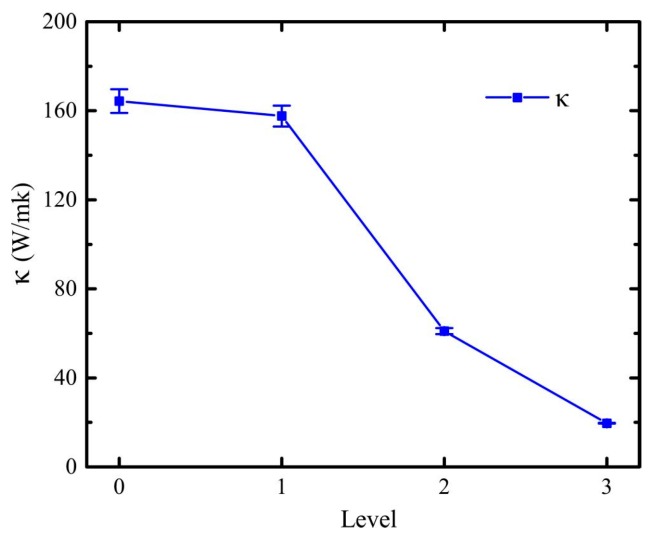
Effect of fractal levels on thermal conductivity of graphene with Sierpinski fractal structures. The cases represent the models of different fractal levels, and the level numbers 0, 1, 2, and 3 represent the structures of gra0, gra1, gra2, and gra3, respectively.

For heterogeneous material, thermal conductivity can be evaluated by the proportion of different components [19,29]. Similarly, the thermal conductivity of graphene with fractal defects was evaluated by Equation (5), of which *∂T*/*∂_X_* is the average of linear fittings in the temperature profiles, as shown in Figure 3. Figure 4 displays the thermal conductivity of graphene with different fractal levels. The simulation results show that the thermal conductivity of the perfect graphene sheet of 21 nm × 21 nm is 164.40 *W m*^−1^ *K*^−1^, which is consistent with the result of the perfect graphene nanoribbon of 19.4 nm × 7 nm [18], 151.60 *W m*^−1^ *K*^−1^. Additionally, the thermal conductivity of fractal graphene decreases with the increase of fractal levels. The thermal conductivity of gra1, 157.62 *W m*^−1^ *K*^−1^, is a little smaller than the value of gra0. The reduction of gra3 thermal conductivity is up to 87.5% of the gra0. As shown in Figure 1, the structure of gra1 is similar with the gra0, except for the defect in the center; as for gra2 and gra3, the defects within interior dramatically increased further. That is, fractal defects alter the thermal transport properties of fractal graphene, but how the defects affect the thermal properties is not clear, especially at the micro level. Usually, the reason for the thermal conductivity reduction of graphene with defects lies in the three main aspects, i.e., the elongation of the real heat flux path, the overestimation of real heat flux area, and the phonon scattering at the edges. In terms of the fractal graphene model structure, the area reduction of heat flux transport is apparent.

**Figure 5:**
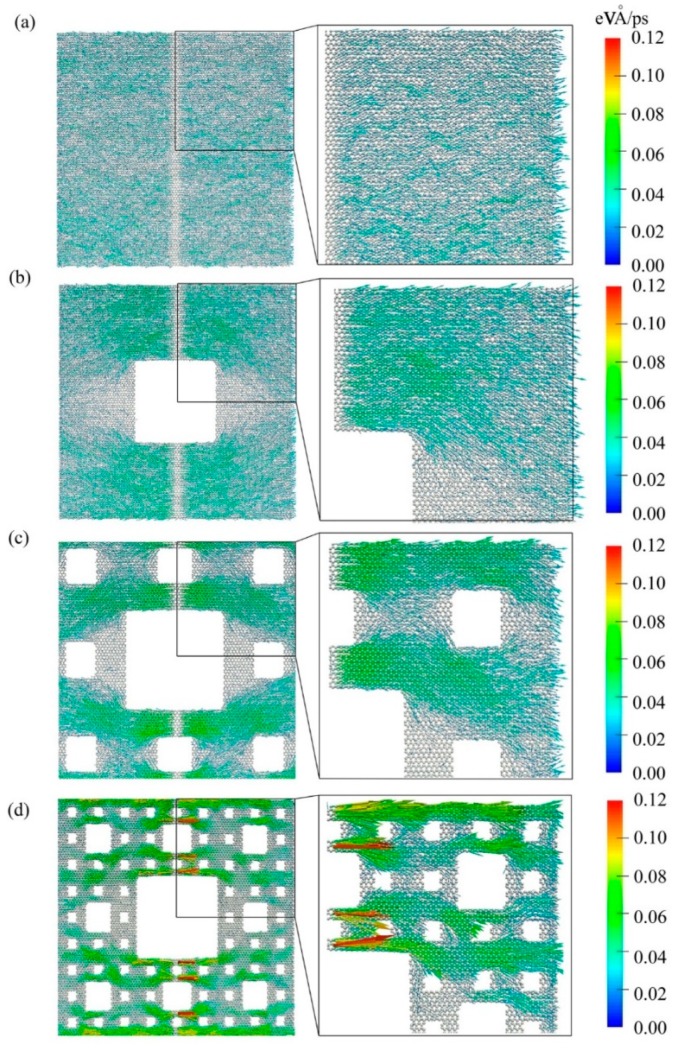
Spatial distribution of heat flux by vector arrows on each atom in fractal graphene in different levels under nonequilibrium steady state. (**a**–**d**) represent the fractal level numbers 0, 1, 2, and 3. The heat flux transport directions are displayed with arrows at graphene with different fractal defects. The zoom in viewport was plotted at the right side.

To gain a better insight, the microheat flux on each atom was calculated by the RNEMD simulations and defined by the expression: *q*_i_ = *e*_i_*v*_i_ − *S*_i_*v*_i_, where *e*_i_, *v*_i_, and *S*_i_ are the energy, velocity vector, and stress tensor of each atom i, respectively [30]. Figure 5 demonstrates vividly the elongation of the heat flux path as well as the phonon scattering in the fractal graphene. The vector arrow shows the migration direction and magnitude of the atomic heat flux. To display the atomic heat flux more clearly, the zoom in viewport is plotted at the right side, and colorful scale also shows the magnitude of the atomic heat flux, where with the color gradually turning to warm, the magnitude increases. 

As shown in Figure 5a, the colors of the atomic heat flux are relatively unvaried, which indicates the migration of the heat flux is a uniform distribution at around 0.02. Moreover, the vectors are generally oriented in the x direction, i.e., the angle of atomic heat flux vectors approaches zero degree with little fluctuations. As shown in Figure 5b–d, with the fractal levels increasing, the color distributions of the atomic heat flux become uneven gradually, and the angles of atomic heat flux vector deviate from the x direction quickly. Furthermore, from Figure 5a–d, some of the vector colors are deepening, which means energy is accumulated in these atoms; while others are fading, which means energy degrades in these atoms. Therefore, the temperature differences at these interfaces grow. This is in line with the enlargement of temperature differences shown in Figure 3a–d. Notably, Figure 5 exhibits directly the real heat flux path. Additionally, the paths of gra1, gra2, and gra3 are longer and longer than that of gra0.

**Figure 6:**
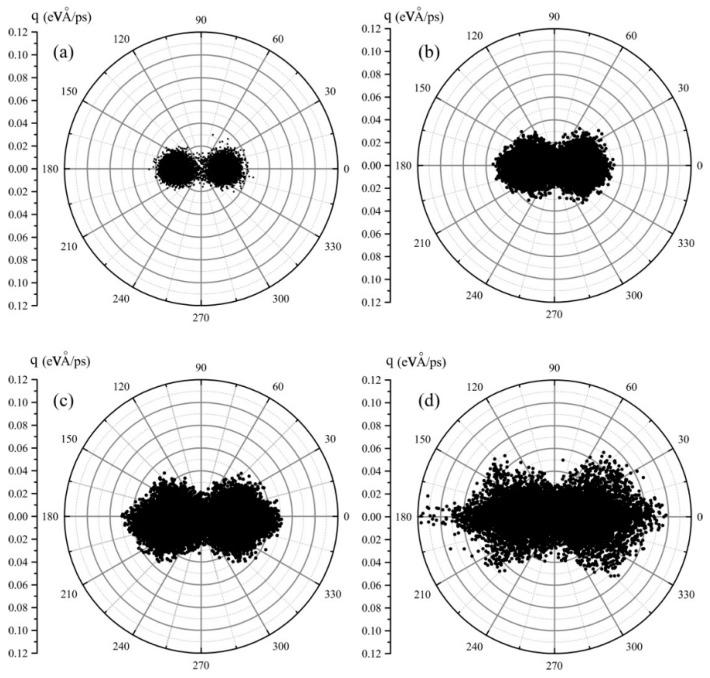
Angle distribution of atomic heat flux vectors in fractal graphene. (**a**), (**b**), (**c**), and (**d**) represent the fractal level numbers 0, 1, 2, and 3.

Although the heat flux transport directions are displayed with arrows at graphene with different fractal defects in Figure 5, the magnitude and angle distribution of the atomic heat flux is perceptual. Thus, we reorganized the atomic heat flux vector data in the form of polar coordinate and replotted them in Figure 6. It shows quantitatively the angle and magnitude distribution of the atomic vector heat flux. As shown in Figure 6a, the angle of the atomic heat flux vector is distributed between 330 to 30 degrees and 150 to 210 degrees, symmetrically. As expected, the angle distribution range of the atomic heat flux vector is wider and wider from Figure 6b–d. As for the shapes of the dotted vectors, from Figure 6a–d, they evolve from two small symmetrical circles to bigger and bigger symmetrical spindles, and the area of the shapes increases with the fractal levels increasing. This implicates that the phonon or heat carriers is scattered dramatically by the defect edges with the increase of fractal levels. In fact, the direction of the atomic heat flux vector can be considered as the average phonon propagation direction. As shown in Figure 6, the phonon scattering occurs qualitatively and quantitatively. However, it is difficult to figure out the magnitude or angle distribution of phonon scattering in statistical analysis because data points overlap in Figure 6. 

**Figure 7:**
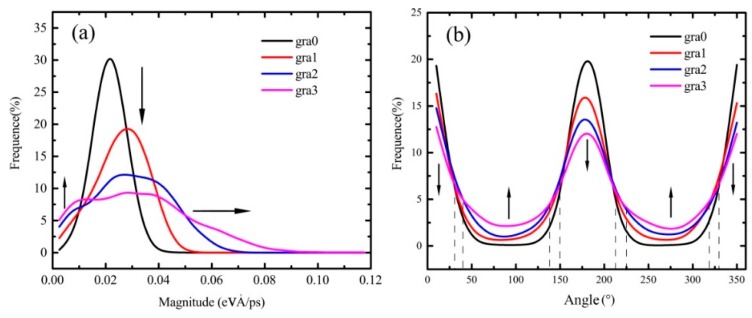
Magnitude and angle distribution of atoms heat flux vectors in fractal graphene statistically. (**a**) Magnitude distribution; (**b**) angle distribution.

Figure 7a exhibits the magnitude distribution of the atomic heat flux vector. The peak values of frequency curves of the atomic heat flux vectors’ magnitude are diminished from gra0 to gra3 with curves flattening. That is, the number of smaller and larger magnitude values increases, while the middle magnitude value decreases. This agrees with Figure 3. The increase of temperature difference contributes to the fractal defect, where the energy accumulation occurs. In addition, the real heat flux path elongates due to the fractal defect, as shown in Figure 5. Figure 7b displays the angle distribution of the atomic heat flux vector. The peak of the frequency curve of the atomic heat flux vectors’ angle diminishes from gra0 to gra3, while the two valleys rise. This indicates that the number of atomic heat flux vectors parallel to the x direction decreases due to the increase of fractal defects, which leads to the phonon scattering, i.e., the propagation direction of the atomic heat flux vector deflects, which is consistent with Figure 5 and Figure 6.

## 5. Conclusions

In this paper, we investigated the thermal transport in length 21 nm × width 21 nm graphene with fractal defects in different levels, gra0, gra1, gra2, and gra3, using the RNEMD method. For gra0, its thermal conductivity is consistent with previous nanoribbon research [18]. For others, their thermal conductivity decreases with the increase of fractal level, and their interfacial thermal resistances are triggered and increase. By calculating and analyzing the atomic heat flux vector, as the fractal level increases, we identified that the reduction of thermal conductivity in graphene with fractal defects was mainly attributed to the elongation of the real heat flux path, the overestimation of the real heat flux area, and phonon scattering at the edge of vacancy, as previous research [18]. However, it is hard to find the quantitative relationship between the elongation of the real heat flux path and the overestimation of the real heat flux area on fractal graphene, for regular and complex edges of fractal geometry. Thus, we made qualitative analyses and a microscopic thermal transportation mechanism of fractal graphene in this work. When fractal defects increase, the edges and boundaries of defects will have a bigger and bigger effect on thermal conductivity, where more and more thermal carriers were trapped and energy accumulated. Meanwhile, the results showed that interfacial thermal resistance increases with the increase of the fractal level. Therefore, by controlling the level of the fractal defect, the thermal properties and communicating capacity of fractal defective graphene have a tradeoff for antennae, which ensures that the fractal graphene antenna could be widely applied to the wireless communication system. 

## Figures and Tables

**Table 1:** Interfacial thermal resistance of different fractal levels.

Levels	∆*T* (K)	R × 10^−11^ (m^2^/W·K)
1	1.920	1.190
2	3.404	3.417
	3.698	3.712
3	7.208	7.554
	11.947	12.520
